# On the use of logarithmic scales for analysis of diffraction data

**DOI:** 10.1107/S0907444909039638

**Published:** 2009-11-17

**Authors:** Alexandre Urzhumtsev, Pavel V. Afonine, Paul D. Adams

**Affiliations:** a IGBMC, CNRS–INSERM–UdS, 1 Rue Laurent Fries, BP 10142, 67404 Illkirch, France; b Physics Department, University of Nancy, BP 239, Faculté des Sciences et des Technologies, 54506 Vandoeuvre-lès-Nancy, France; c Lawrence Berkeley National Laboratory, One Cyclotron Road, BLDG 64R0121, Berkeley, CA 94720, USA; d Department of Bioengineering, University of California Berkeley, Berkeley, CA 94720, USA

**Keywords:** resolution, logarithmic scale, *R* factor, data-to-parameter ratio

## Abstract

Predictions of the possible model parameterization and of the values of model characteristics such as *R* factors are important for macromolecular refinement and validation protocols. One of the key parameters defining these and other values is the resolution of the experimentally measured diffraction data. The higher the resolution, the larger the number of diffraction data *N*_ref_, the larger its ratio to the number *N*_at_ of non-H atoms, the more parameters per atom can be used for modelling and the more precise and detailed a model can be obtained. The ratio *N*_ref_/*N*_at_ was calculated for models deposited in the Protein Data Bank as a function of the resolution at which the structures were reported. The most frequent values for this distribution depend essentially linearly on resolution when the latter is expressed on a uniform logarithmic scale. This defines simple analytic formulae for the typical Matthews coefficient and for the typically allowed number of parameters per atom for crystals diffracting to a given resolution. This simple dependence makes it possible in many cases to estimate the expected resolution of the experimental data for a crystal with a given Matthews coefficient. When expressed using the same logarithmic scale, the most frequent values for *R* and *R*_free_ factors and for their difference are also essentially linear across a large resolution range. The minimal *R*-factor values are practically constant at resolutions better than 3 Å, below which they begin to grow sharply. This simple dependence on the resolution allows the prediction of expected *R*-factor values for unknown structures and may be used to guide model refinement and validation.

## Abstract 2

Conventional and free *R* factors and their difference, as well as the ratio of the number of measured reflections to the number of atoms in the crystal, were studied as functions of the resolution at which the structures were reported. When the resolution was taken uniformly on a logarithmic scale, the most frequent values of these functions were quasi-linear over a large resolution range.

## 1. Introduction   

The maximum resolution of diffraction is an important characteristic of experimental data sets and the resulting crystallo­graphic Fourier synthesis maps. The number of structure factors *N*_ref_ for a given crystal depends on the resolution *d* as
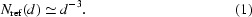
Binning of diffraction data, *e.g.* for the reporting of statistics, can be chosen to be uniform in Å, in sin(θ)/λ, in Å^−1^, Å^−2^, Å^−3^ *etc*. For example, if the resolution limits *d*_*k*_, *k* = 1, 2, …, are chosen uniformly in Å^−3^,
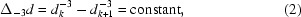
moving from *d*_*k*_ to *d*_*k*+1_ changes the number of reflections approximately by the same amount for all *k*, *i.e.* equal volumes of reciprocal space are covered by each bin. Here, we analyze the effects of partitioning *d*_*k*_ uniformly using a logarithmic scale,
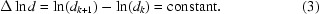
In this case, moving from *d*_*k*_ to *d*_*k*+1_ changes the number of reflections by approximately the same factor. Using this regime, we can perform analyses to establish whether selected crystallographic characteristics have a simple dependence on resolution on this logarithmic scale. One such characteristic is the ratio of the number of diffraction data *N*_ref_ to the number *N*_at_ of atoms for structures solved at a given resolution. Ideally, the total number of parameters of a model should not exceed the number of independent observations (reflections) or the model is considered to be overparametrized and inappropriate for refinement. Therefore, the typical value of *N*_ref_/*N*_at_ at a given resolution indicates the allowed number of parameters per atom and therefore defines a ‘typical model’ at this resolution. Knowledge of this ratio can also help to predict the number of molecules per unit cell. Inversely, for a known Matthews coefficient (Matthews, 1968 ▶),
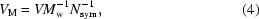
it may help to estimate the expected high-resolution diffraction limit of the crystal as discussed below, thus completing other indicators (see, for example, Arai *et al.*, 2004 ▶, and references therein), in particular the overall *B* value (Wilson, 1949 ▶). Here, *V* is the unit-cell volume, *N*_sym_ is the number of crystallographic symmetry operations and *M*_w_ is the molecular weight of the macromolecules in the asymmetric part of the unit cell.

Expected ‘typical’ values of the crystallographic *R* factor, of the *R*_free_ value (Brünger, 1992 ▶) and of their difference are often considered during structure solution. To our knowledge, despite numerous studies (for example, Luzzati, 1952 ▶; Cruickshank, 1996 ▶; Brünger, 1997 ▶; Tickle *et al.*, 1998 ▶, 2000 ▶; Read & Kleywegt, 2009 ▶; Urzhumtseva *et al.*, 2009 ▶; Joosten *et al.*, 2009 ▶), a convenient and simple analytic expression for the *R* factors typical at a given resolution is still not well defined. We used a logarithmic scale to study these functions and also the minimal values of the *R* factor. The latter can be considered as a goal that in most cases can be achieved at a given resolution.

Summarizing, the goal of this study was to determine whether an appropriate choice of resolution binning using different scales highlights a simple analytic dependence of macromolecular model characteristics. Knowledge of such a dependence can help in structure solution and can be used as an auxiliary validation criterion.

## 2. Test data and parameters   

We selected models from the PDB (Bernstein *et al.*, 1977 ▶; Berman *et al.*, 2000 ▶; selection in March 2009) for which the database contained experimental data: 31 662 entries in total (set 1). For these models we extracted the characteristics as they were reported in the file headers. Two subsets (sets 2 and 3), with 29 484 and 710 entries, respectively, consisted of models of proteins only and models that included nucleic acids.

Independently, a number of crystallographic characteristics, including *R* factors, were recalculated using the *phenix.model_vs_data* (Afonine *et al.*, in preparation) utility of *PHENIX* (Adams *et al.*, 2002 ▶). Set 4 consisted of 30 546 entries, which were those of set 1 excluding obvious outliers as indicated by *R* factor. Set 5 consisted of entries for which a test set was available allowing the calculation of *R*_free_ factors and con­tained 22 504 entries in total. Details of these data sets are given below.

For our uniform logarithmic grid we needed to define its step and origin. We chose the step Δln*d* such that from one resolution limit to another the number of reflections changed by a factor of 1.5. [It follows from equations 1 and 3 that Δln*d* = 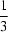
ln(1.5) ≃ 0.135.] Also, for convenience of presentation we chose the origin *d*_1_ = 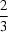
Å such that the resolution *d* = 1.0 Å (ln*d* = 0.0) falls exactly at a grid node.

## 3. Number of data per atom   

### 3.1. Preliminary analysis for selected data sets   

As mentioned above, the ratio *N*_ref_/*N*_at_, the ratio of the number of independent reflections *N*_ref_ to the number *N*_at_ of independent macromolecular non-H atoms in the unit cell, is important in helping to define the possible parameterizations of an atomic model when working with diffraction data at a given resolution. The total number of reflections at a given resolution *d* can be expressed through the volumes *V* and V* of the unit cell in direct or reciprocal space, respectively, as
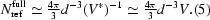
When the structure factors obey Friedel’s law, for a given crystal the dependence on resolution is
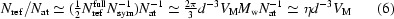
(otherwise the coefficient ½ would be absent). For protein structures, the mean ratio *M*_w_ *N*_at_^−1^ can be approximately estimated from the molecular weight and atom content of different residues, resulting in the coefficient η = (2π/3)*M*_w_ *N*_at_^−1^ ≃ 27.

**Figure 1:**
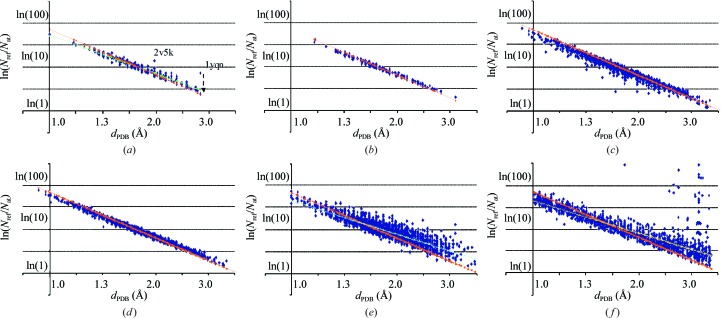
Distribution of the ln(*N*_ref_/*N*_at_) value *versus* resolution *d*_PDB_ on a uniform logarithmic scale. (*a*) Structures with Matthews coefficient 2.35 < *V*_M_ < 2.45 Å^3^ Da^−1^. *V*_M_ is taken from the file headers and data completeness is above 99%. The broken arrow shows the change in the ratio after a correct assignment of *N*_at_ for 1yqn. (*b*) The same as (*a*) but with *V*_M_ recalculated. (*c*) The same as (*b*) but without selection of entries by data completeness. (*d*) The same as (*c*) but with correction for data completeness. (*e*) All models with data completeness above 99%. (*f*) Random selection from the whole PDB with correction for data completeness. The orange line corresponds to theoretical values for crystals with *V*_M_ = 2.4 Å^3^ Da^−1^. The green, blue and yellow lines show the linear approximations for (*a*), (*e*) and (*f*), respectively. See text for details.

We calculated the ratio *N*_ref_/*N*_at_ for all models of set 1. Here, *N*_at_ is the number of non-H atoms in the PDB model and *N*_ref_ is equal to the number of reflections in the deposited file; anomalous pairs of reflections, which are highly correlated, were considered as a single reflection when presented (in 1051 data sets). In our study, we characterize the structure by the resolution *d*_PDB_ at which the deposited structure has been reported. Obviously, this characteristic depends on a number of subjective factors such as the accepted completeness of the highest resolution zone, particular experimental conditions and restrictions *etc*. However, the large number of structures available from the current PDB for our analysis minimizes any systematic bias arising from these factors. Our first goal was to determine whether the dependence of the calculated *N*_ref_/*N*_at_ and reported *d*_PDB_ reflects relation (6). Fig. 1 ▶(*a*) shows the distribution of ln*N*_ref_/*N*_at_ *versus* resolution *d*_PDB_ on a uniform logarithmic scale for a subset of models with data completeness above 99% and a Matthews coefficient of 2.35 < *V*_M_ < 2.45 Å^3^ Da^−1^, close to the typical value for *V*_M_ of 2.4 Å^3^ Da^−1^. *V*_M_ was taken from the PDB headers; the selection gave 313 models. The points fitted well to a straight line. Two obvious outliers correspond to the models 2v5k and 1yqn, for which the deposited atoms correspond to one half and one third of the whole cell content, respectively, owing to corresponding local (noncrystallographic) symmetries. When these symmetries were taken into account, the points fitted closely to the line (see, for example, the case of 1yqn indicated by an arrow in Fig. 1 ▶ *a*).

The slope of the straight line differs slightly from −3 as expected from (6). We supposed that some differences might be found in the reported *V*_M_ values. For example, at high resolution some authors may include H atoms, differently from at lower resolutions; conversely, at low resolutions one might miss the contribution of disordered parts or side chains that are invisible in maps and absent from the model. To study this issue, we recalculated the *V*_M_ value for all reported structures considering the full macromolecular content of the cell according to the deposited sequence. Obviously, this recalculation modified the set of selected models (291 models with 2.35 < *V*_M-calc_ < 2.45 Å^3^ Da^−1^).

When the PDB-reported *V*_M_ values were substituted by the recalculated values, the plot of ln*N*_ref_/*N*_at_ had the expected slope (Fig. 1 ▶ *b*). This observation also gave us confidence that there was no significant discrepancy between the resolution limits *d*_PDB_ in the PDB-reported structures and that further analysis could be based on these values.

A similar ln*N*_ref_/*N*_at_ *versus d*_PDB_ distribution for all models with 2.35 < *V*_M-calc_ < 2.45 Å^3^ Da^−1^ (Fig. 1 ▶ *c*; 2754 models) con­tains several points that are below this line owing to in­complete data sets. The data completeness ‘compl’ was then taken into account so that in further calculations *N*_ref_ corresponded to a complete set of data as measured at a reported resolution *d*_PDB_, *N*_ref_^full^ = *N*_ref_compl^−1^. This new distribution (Fig. 1 ▶ *d*) has the same features as that in Fig. 1 ▶(*b*) but is more significant statistically. In general, correcting for completeness instead of rejecting models with incomplete data sets makes the set of models much more representative. In particular, crystals with strongly anisotropic diffraction patterns can be studied together with isotropically diffracting structures with no need for the introduction of artificial selections.

When we analyze the distribution of ln*N*_ref_/*N*_at_ for all PDB entries with compl > 99% we observe that the corresponding cloud of points is larger but still essentially linear (Fig. 1 ▶ *e*; 4020 models). However, the slope of the principal axis is now significantly lower than previously calculated. Kantardjieff & Rupp (2003 ▶) studied the dependence of *V*_M_ on different factors and in particular showed that the mean *V*_M_ increases with resolution; according to (6) this explains the lower slope we observed. An alternative calculation without selection by compl > 99% but using the completeness-corrected number of reflections *N*_ref_^full^ as above showed a similar distribution (Fig. 1 ▶ *f*; for illustration purposes we selected randomly 250 models per resolution shell; shells with less than 200 models were excluded; 2489 models in total).

### 3.2. Maximum–mean–minimum analysis   

To analyze the features of the distributions obtained in §[Sec sec3.1]3.1, we studied them in more detail as described below. Our goal was to find a simple dependence of the principal statistical characteristics of *N*_ref_/*N*_at_ as a function of resolution. Following Kantardjieff & Rupp (2003 ▶), in order to work with a more homogenous set of models we excluded all entries containing nucleic acids. This left us with 29 486 entries (set 2; Table 1 ▶). In order to have a sample size that was as large as possible we did not reject incomplete data sets but, in accordance with preliminary analysis, used the completeness-corrected values of *N*_ref_ as above.

Table 2 ▶ shows the average and maximal values of the ratio *N*_ref_/*N*_at_ in different resolution shells. In a number of shells the maximal value exceeds the average values more than the variation of the Matthews coefficient would allow according to (6). This happens often for crystals with a high local symmetry, in particular for crystals of viruses. One reason is the presence of coordinates for only one molecule of several linked by a local symmetry, similar to the 2v5k and 1yqn cases (see §[Sec sec3.1]3.1). Another reason is missed atoms in disordered parts or domains. We choose not to eliminate or correct these structures as to do so could involve multiple subjective choices.

**Figure 2:**
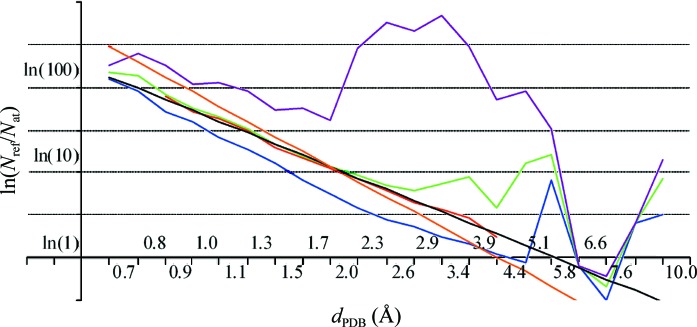
Logarithm ln(*N*_ref_/*N*_at_) as a function of resolution *d*_PDB_ on a uniform logarithmic scale. The curves show the minimal (blue), maximal (violet), average (green) and mode (red) values for the protein structures reported in the PDB (set 2). The mode line is shown as the interval in which this value was calculated. The straight line in orange is the same as in Fig. 1 ▶ showing the ratio for crystals with *V*_M_ = 2.4 Å^3^ Da^−1^. The black line shows the linear interpolation to the mode.

The logarithm of the minimal ratio *N*_ref_/*N*_at_ for resolutions up to 2.5 Å closely follows the line with slope equal to −3 (Fig. 2 ▶). Corresponding crystals have a *V*_M_ (2) close to 1.5 Å^3^ Da^−1^. For comparison, Fig. 2 ▶ also shows the straight line for crystals with *V*_M_ = 2.4 Å^3^ Da^−1^, as in Fig. 1 ▶.

Fig. 2 ▶ also shows that at resolutions greater than 2.5 Å the logarithm of the average value 〈*N*_ref_/*N*_at_〉 is a quasi-linear function of the logarithm of the resolution, ln*d*_PDB_. As expected from Fig. 1 ▶, the slope of this line differs from those of the lines corresponding to the *V*_M_ constant. This agrees with the previous demonstration by Kantardjieff & Rupp (2003 ▶) that on average the lower the resolution of the crystals, the larger the Matthews coefficient [these authors also made a linear regression analysis for *V*_M_(*d*_PDB_) using an intuitive resolution scale]. Table 3 ▶ gives the coefficients of the corresponding linear approximation performed in the interval (0.8 Å, 2.6 Å) and the r.m.s.d. (root-mean-square deviation) from it. One can observe that for a few structures reported with an upper diffraction limit of between 5.8 and 7.6 Å the points for their 〈*N*_ref_/*N*_at_〉 also fall on this line.

### 3.3. Studies of the mode   

Outliers with a very large *N*_ref_/*N*_at_ may influence the 〈*N*_ref_/*N*_at_〉 values. For example, 〈*N*_ref_/*N*_at_〉 significantly fluctuates at low resolution (see discussion above). At the same time, the other characteristics of a distribution such as the values of the most frequent *N*_ref_/*N*_at_ for a given resolution, the mode μ(*N*_ref_/*N*_at_), are much less sensitive to outliers.

**Figure 3:**
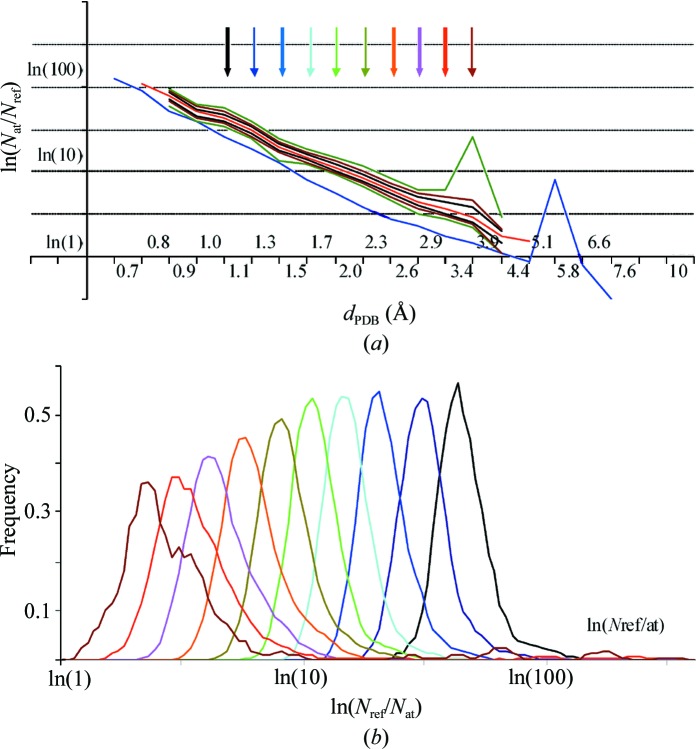
(*a*) The mode μ(*N*_ref_/*N*_at_) as a function of the resolution *d*_PDB_ on a uniform logarithmic scale. The thick red curve shows the mode values as a function of resolution on a uniform logarithmic scale for the protein structures reported in the PDB (set 2). The thin lines show, as corridors, the distribution of the models around the mode. Each corridor contains 40% (black), 60% (brown) and 80% (dark green), respectively, of the structures in the corresponding resolution shell, half above and half below the mode. The corridors are shown at a resolution interval with a high enough number of models to calculate these values; the mode was formally calculated and is also shown for one higher resolution interval and one lower resolution interval even when the statistics there were poor. The blue line shows the minimal values for comparison (Table 1 ▶). Coloured arrows correspond to the distributions shown in (*b*). (*b*) Distribution of *N*_ref_/*N*_at_ for several selected resolutions as indicated by coloured arrows in (*a*).

For resolution shells better than 0.8 Å or worse than 4.4 Å the number of available structures is low and thus the statistics are relatively poor. For other shells the distribution of *N*_ref_/*N*_at_ is essentially unimodal, with a relatively symmetric peak for the most frequent values (Fig. 3 ▶; see also the relevant Fig. 3 ▶ in Kantardjieff & Rupp, 2003 ▶). In the resolution shells between approximately 0.9 and 2.5 Å the mode μ(*N*_ref_/*N*_at_) essentially coincides with 〈*N*_ref_/*N*_at_〉 (Fig. 2 ▶). For lower resolutions of up to 4.4 Å 〈*N*_ref_/*N*_at_〉 deviates from the straight line while the mode μ(*N*_ref_/*N*_at_) continues following it. In fact, even in the intervals with relatively poor statistics, 4.4–5.1 and 0.67–0.76 Å, the most frequent values of *N*_ref_/*N*_at_ also follow this straight line (Fig. 3 ▶, Table 3 ▶).

The corresponding linear interpolation (Table 3 ▶) allows the ‘most typical *N*_ref_/*N*_at_ value at a given resolution’ to be estimated analytically as
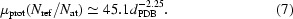
Table 2 ▶ shows interpolated and extrapolated values together with experimentally obtained values.

For crystals of nucleic acids without proteins the behaviour is quite similar (details not shown) even though the statistics are much poorer owing to the small sample size (set 3; Table 1 ▶). The linear approximation of the mode μ_nucl_(*N*_ref_/*N*_at_), 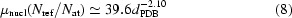
differs only slightly from that obtained for proteins (Table 3 ▶).

### 3.4. Possible applications   

This simple behaviour of typical *N*_ref_/*N*_at_ values over a wide resolution range may be helpful for existing tools, for example *Matthews Probability Calculator* (Kantardjieff & Rupp, 2003 ▶) or *phenix.xtriage* (Zwart *et al.*, 2005 ▶), especially at extreme resolutions. Com­bining (6) and (7) gives a simple analytic estimation
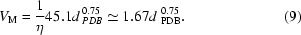
Inverting (9), one can estimate the limit
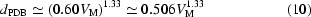
to which a crystal with a given *V*_M_ is expected to diffract. This information could be taken into account when con­sidering how much effort should be applied to obtaining improved diffraction data from a given crystal form of a specific protein. Obviously, (10) only provides a typical limit, while better results may be obtained for a particular crystal. As an example, human aldose reductase crystals have a *V*_M_ of 2.10 Å^3^ Da^−1^, giving an estimated *d*_PDB_ of ∼1.35 Å. This confirms that the value of 1.7 Å initially reported at a home source (Lamour *et al.*, 1999 ▶) was below what might be obtained. At the same time, (10) does not predict that some aldose reductase crystals can diffract to 0.66 Å resolution (Howard *et al.*, 2004 ▶). Nevertheless, the possibility of similarly high-resolution data can be predicted for other crystals. An example is the polypeptide YGG crystal (Pichon-Pesme *et al.*, 2000 ▶; *V*_M_ = 1.12 Å^3^ Da^−1^) for which (10) gives *d*_PDB_ ≃ 0.60 Å. Indeed, for this crystal the 50% completeness data set was measured at 0.59 Å resolution (the highest resolution reflection measured was at 0.44 Å resolution).

The predictability of the typical *N*_ref_/*N*_at_ values suggests the definition of the maximal number of parameters per atom that are ‘usual at a given resolution’, avoiding overparametrization (Table 2 ▶). In other words, this defines the number of atomic parameters that can typically be used at a given resolution. While for a particular model the number *N*_ref_/*N*_at_ can be calculated precisely at any given resolution, knowledge of typical values is crucial for software and methods developers, allowing them to automate model-refinement protocols. In particular, the ratios of 4 and 10 at resolutions of approximately 3 and 2 Å, respectively, give the minimal theoretical limits at which individual isotropic or anisotropic displacement parameters can be used (with four or ten parameters per atom, respectively). Obviously, in these cases the ratio *N*_ref_/*N*_at_ ≃ 1 and therefore in practice higher resolution limits are recommended even when various restraints are introduced. The possibility of unrestrained refinement is not surprising at 1 Å or higher, where there are four reflections per parameter even for an anisotropic model. A very high ratio of above 80 at resolutions better than 0.8 Å leads one to believe that the diffraction data will contain a lot of additional information (as confirmed by residual maps) and that a more detailed model is required. At the low-resolution end, the typical ratio prescribes the size of rigid groups that can realistically be introduced.

## 4. *R* factors on a logarithmic scale   

### 4.1. PDB-reported *R* factors   

While *N*_ref_/*N*_at_ characterizes the amount of ‘diffraction information’ at a given resolution and defines the type of model, the crystallographic *R* factor is a conventional measure of the diffraction quality of these models, although it is not fully reliable as indicated in a series of papers starting with Brändén & Jones (1990 ▶). There are anecdotal ‘rules of thumb’ for acceptable values. We searched for a simple dependence of *R* factors on the resolution, substituting the usual uniform resolution scale by a uniform logarithmic scale.

For our analysis we took the same full set of 31 662 models (set 1) as above. We excluded 1088 entries with an incorrectly reported value of the *R* factor (*R*_PDB_). We also removed 15 structures with *R*_PDB_ > 17.0 (probably reported as a percentage and not as a fraction) and 11 models for which the reported *R*_PDB_ represented values other than the conventional *R* factor (for all these entries the value was below 0.06). For other entries, excluding a nonmacromolecular model of actino­mycin (PDB code 1a7y; Schäfer *et al.*, 1998 ▶; *R*_PDB_ = 0.058), the reported value *R*_PDB_ varied between 0.072 and 0.615. Exluding actinomycin, we arrived at a total of 30 546 models (set 4; Table 1 ▶).

**Figure 4:**
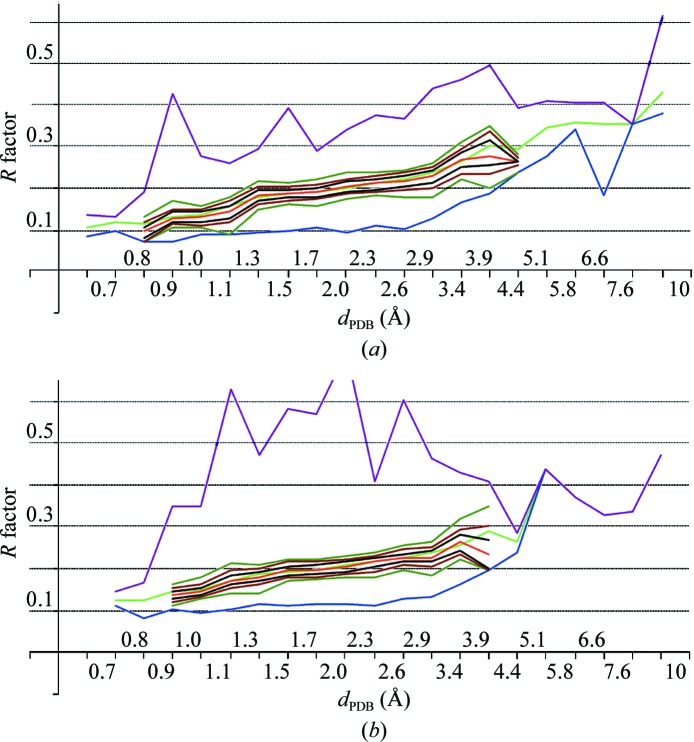
*R* factors as a function of resolution *d*_PDB_ on a logarithmic scale. The curves show the minimal (blue), average (green), maximal (violet) and mode (red) values; the mode is calculated in the intervals containing a high enough number of models. The thin lines show the corridors around the mode. Each corridor contains 40% (black), 60% (brown) and 80% (dark green) of the structures, respectively, in the corresponding resolution shell, half above and half below the mode. (*a*) *R* factors reported in the PDB; set 4 of models. (*b*) *R* factors recalculated with *phenix. model_vs_data*; set 5 of models.

The same resolution intervals with an equal length on the logarithmic scale were used as defined in §[Sec sec2]2. Resolution shells at very high and low resolutions had poor statistics. In each of the other resolution shells the distribution of *R* factors was uni­modal, with a clear value for the mode μ(*R*_PDB_). In all shells up to the resolution shell 3.0–3.5 Å the peaks were more or less symmetric and quite narrow. The intervals [μ(*R*_PDB_) − δ, μ(*R*_PDB_) + δ] contained nearly 40, 60 or 80% of the structures reported at this resolution *d*_PDB_ when δ = 0.01, 0.02 or 0.03, respectively (Fig. 4 ▶ *a*). Where calculated, μ(*R*_PDB_) is close to the average value 〈*R*_PDB_〉.

**Figure 5:**
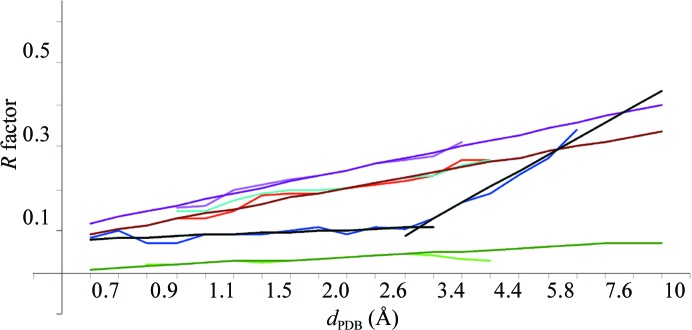
Linear approximation to the *R* factors. The red and blue curves show the mode and minimal values for the *R* values extracted from the PDB headers. The curves in magenta and in green show the mode value for the *R*_free_ factor and for the difference factor Δ*R* = *R*_free_ − *R* recalculated for set 5 of models. The straight lines in brown, black, violet and dark green illustrate the corresponding linear approximations (Table 3 ▶). The line in light blue shows the mode for the *R* factor recalculated for the largest possible set of models (set 4). The curves are shown for resolution shells containing a high enough number of models to calculate the values.

It is has previously been observed that 〈*R*_PDB_〉 increases with resolution and that this growth is nonlinear on a uniform scale in angstroms (see, for example, Read & Kleywegt, 2009 ▶; Joosten *et al.*, 2009 ▶). However, it is practically linear up to 3.5 Å when the resolution is expressed on the logarithmic scale, as is μ(*R*_PDB_) (Fig. 5 ▶). Table 3 ▶ gives the coefficients of the corresponding linear interpolations (Table 4 ▶). The r.m.s.d. of the interpolation
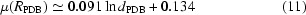
in the interval (0.87, 3.86) does not change on including μ(*R*_PDB_) values for lower and higher resolution intervals with poorer statistics.

Interestingly, the minimal values *R*_PDBmin_ are practically constant at around 0.10 in all resolution shells up to 2.6 Å (Fig. 4 ▶ *a*). In other words, at all these resolutions it is possible to obtain a conventional atomic model reproducing the experimental diffraction data with a similar and sufficiently small relative error (*R* factor). The approach of μ(*R*_PDB_) and 〈*R*_PDB_〉 to 0.10 at near-atomic resolutions of ∼1 Å and the statistically significant number of reported models means that here most of the models achieve this high quality. The increase in μ(*R*_PDB_) with resolution from 1 to 3 Å indicates that while it is still possible to obtain a high-quality model, this requires more and more high-quality data, particular effort and luck. Below 2.6 Å resolution *R*_PDBmin_ starts to grow sharply. At a similar resolution, the minimal Matthews coefficient of known macromolecular crystals also starts growing as indicated by changing the slope of the curve min ln(*N*_ref_/*N*_at_) (Fig. 2 ▶).

In §[Sec sec5]5 we speculate about the possible meaning of the intersection of the straight lines for 〈*R*_PDB_〉 and μ(*R*_PDB_) with the curve for *R*_PDBmin_ at resolutions of ∼0.7–0.8 Å and ∼6 Å.

### 4.2. Recalculated *R* factors   

In order to remove errors and inconsistencies in *R*_PDB_ other than those indicated above in §[Sec sec4.1]4.1, we recalculated the *R*-­factor value for all 32 662 structures using the *phenix.model_vs_data* tool of *PHENIX*. Extremely high or unreasonably low values of the calculated *R* factor indicated some inconsistency between the reported models or data. In spite of these obvious outliers, the general behaviour of the *R* factor was similar to that for *R*_PDB_ [details not shown; see Fig. 5 ▶ for the mode μ(*R*) values]. For some models the obtained *R* values were slightly higher than *R*_PDB_, while for others they were lower. The details of this comparison will be reported elsewhere. In general, the average difference is within reasonable limits. It is slightly positive at higher resolutions (*d*_PDB_ < 1.2 Å), where for a number of models it was impossible to reproduce accurately the authors’ calculations.

We chose not to remove outliers using σ or outlier cutoff levels, the choice of which is subjective. Instead, we repeated the calculations with a subset containing the entries for which the test data sets were available and the *R*_free_ value could be calculated (set 5; 22 504 models). Here, all models had 0.082 ≤ *R* ≤ 0.626, with a single exception (*R* = 0.715); thus, outliers did not strongly influence the average and especially the mode values (Fig. 4 ▶ *b*).

Qualitatively, the behaviour of the *R* factor for both sets of models (sets 4 and 5) is similar to that of *R*_PDB_. For the recalculated *R* factors, which are unbiased by the diversity of protocols and software, the mode μ(*R*) is a quasi-linear function of ln*d*_PDB_ in the whole resolution range in which it was calculated (up to 4.4 Å). For the reasons mentioned above this line has a slope that is slightly lower (Table 3 ▶) than that for μ(*R*_PDB_).

### 4.3. *R*_free_ and difference *R*_free_ − *R*   

In general, the *R*_free_ calculated for set 5 of the PDB entries behaved similarly to *R*. On the logarithmic scale 〈*R*_free_〉 is quasi-linear up to a resolution of 4 Å. The same was observed for μ(*R*_free_) in all intervals in which it was possible to calculate it (Fig. 5 ▶). Table 3 ▶ gives the coefficients of the corresponding linear approximation (Table 4 ▶).

The difference Δ*R* = *R*_free_ − *R*, which is useful for model validation, is on average positive as expected (Brünger, 1992 ▶). All resolution shells contained obvious outliers with Δ*R* close to 0 or even negative. The mode values μ(Δ*R*) are independent of these outliers and therefore we did not exclude them by subjective cutoffs. These characteristics are practically linear at resolutions higher than 3 Å (Fig. 5 ▶). This makes it possible to suggest a simple formula for the Δ*R* typical at a given resolution *d*_PDB_ (Table 3 ▶),
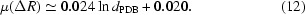
At resolutions below 3 Å the difference μ(Δ*R*) is lower than[Disp-formula fd12] that predicted by (12). On one hand, there is no proof that (12) should be applicable at all resolutions. On the other, there are a number of hypothetical reasons that could decrease the reliability of *R*_free_ statistics for low resolutions. For example, a smaller number of reflections may make test sets and corresponding statistics poorer, reflections from the test sets may be indirectly related to those from the work sets for structures with local symmetries (Fabiola *et al.*, 2006 ▶; as discussed in §[Sec sec3.2]3.2, such structures are more frequent at lower resolutions) *etc*.

## 5. Discussion   

A nonlinear rescaling of a function or its argument(s) modifies the shape of its plot and a judicious choice of scale may help to clarify the dependence. Obviously, the simplest dependence is a linear dependence, which can even be identified visually. In crystallo­graphy, many characteristics are functions of resolution. The resolution scale is usually linear, quadratic or cubic, either in direct or in reciprocal space, or chosen in some other intuitive way. The logarithmic scale we have described naturally increases the number of reflections by a given factor from one resolution limit to another when the limits are chosen uniformly. In our study we have analyzed several crystallo­graphic characteristics as a function of the resolution *d*_PDB_ at which structures have been reported. In contrast to traditional studies of the mean values of functions, we analyzed their modes μ (most frequent values), which are less sensitive to outliers, although in many cases the conclusions are also applicable to the mean values.

The ratio *N*_ref_/*N*_at_ of the number of independent reflections to the number of independent macromolecular non-H atoms in the unit cell is an important characteristic of structural projects. It is an appropriate candidate for study using a logarithmic scale because of the cubic dependence of *N*_ref_/*N*_at_ on *d*_PDB_ for crystals with the same Matthews coefficient. A derived dependence of μ(*N*_ref_/*N*_at_) on *d*_PDB_ with a power close to −2.2 was easily observed when using the logarithmic scale and is difficult to deduce otherwise. This dependence can be used to help define the upper limits on the parameterization of macromolecule models possible at a given resolution. It may also be used to help to predict the number of molecules in the unit cell or to estimate the expected diffraction limit of a crystal.

Using a logarithmic scale to study *R* factors is less intuitive. However, in contrast to previous studies using traditional scales, here quasi-linear behaviour was observed for the mode of *R* factors both reported in the PDB and recalculated from the models and data. Similarly, the mode for *R*_free_ and the difference between *R* factors are linear at resolutions better than 3 Å. Corresponding linear approximations can be used to help to guide refinement and validation of atomic models.

Interestingly, the two points of the intersection of the straight line for μ(*R*) with the curve for *R*_min_ have common features. They both mark limits where correcting terms to the structure factors of a conventional independent-atoms model (*F*_IAM_),
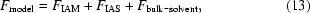
become crucial: a bulk-solvent contribution *F*_bulk-solvent_ (see, for example, Jiang & Brünger, 1994 ▶) below the low-resolution limit of ∼6 Å and density-deformation structure factors *F*_IAS_ (for example, using interatomic scatterers; Afonine *et al.*, 2004 ▶) at ultrahigh resolution, *i.e.* higher than approximately 0.7 Å. Efficient bulk-solvent (Afonine *et al.*, 2005 ▶) and IAS corrections (Afonine *et al.*, 2007 ▶) are available in *PHENIX*. We conclude that these resolution extremes mark points at which features of the electron density are not well modelled by single isotropic or anisotropic scatterers centred on the atomic positions.

We postulate that other crystallographic phenomena can be uncovered using a uniform logarithmic scale. For example, the peak distribution in the averaged and individual |*E*(*d*)| profiles (Morris & Bricogne, 2003 ▶; Morris *et al.*, 2004 ▶) is more or less uniform when using a logarithmic scale. However, at present we cannot determine whether this is purely coincidental or the result of some underlying physical meaning.

## Figures and Tables

**Table 1:** Number of models in different sets used for statistics Columns 3 and 4 show the median of the intervals in angstroms and on a logarithmic scale. See text for descriptions of the data sets.

*N*	Resolution shell (*d*_1_ *d*_2_) ()	Median (*d*_1_ *d*_2_)^1/2^ ()	ln-median ln(*d*_1_ *d*_2_)	Set 1 (with *F*_obs_)	Set 2 (no nucleic acids)	Set 3 (nucleic acids)	Set 4 (17.0 > *R*_PDB_ > 0.06)	Set 5 (with test data set)
1	0.67			3	3	0	3	0
2	0.670.76	0.71	0.338	8	7	1	7	3
3	0.760.87	0.82	0.206	42	41	1	38	12
4	0.871.00	0.93	0.070	196	178	16	177	76
5	1.001.14	1.07	0.066	336	312	22	319	167
6	1.141.31	1.22	0.201	729	687	34	687	432
7	1.311.50	1.40	0.338	1878	1794	67	1807	1230
8	1.501.72	1.61	0.474	3639	3459	119	3470	2592
9	1.721.97	1.84	0.610	6574	6329	90	6248	4676
10	1.972.25	2.11	0.744	7169	6790	132	6916	5211
11	2.252.58	2.41	0.879	5385	4992	103	5256	4001
12	2.582.95	2.76	1.015	3821	3355	84	3767	2823
13	2.953.37	3.15	1.148	1451	1222	37	1428	1039
14	3.373.86	3.61	1.283	310	232	4	308	190
15	3.864.42	4.13	1.418	82	64	0	78	44
16	4.425.06	4.73	1.554	16	9	0	15	3
17	5.065.80	5.42	1.690	6	3	0	5	1
18	5.806.63	6.20	1.825	5	1	0	5	1
19	6.637.59	7.09	1.959	6	5	0	6	1
20	7.598.69	8.12	2.095	1	1	0	1	1
21	8.699.95	9.30	2.230	5	2	0	5	1
	Total			31662	29486	710	30546	22504

**Table 2:** Statistical information for *N*_ref_/*N*_at_ in the resolution shells chosen uniformly on a logarithmic scale Columns 2 and 3 give the PDB codes for the protein structures with the minimal and maximal value of the ratio. Columns 7 and 8 show the values of the linear interpolations in the resolution interval (0.76, 2.58) (see Table 3 ▶). The last column gives the difference of the modes calculated for sets 1 and 2 of the models.

	PDB code		*N*_ref_/*N*_at_			Linear interpolation		
Resolution shell ()	Min. *N*_ref_/*N*_at_	Max. *N*_ref_/*N*_at_	Min.	Max.	Mean	Mean	Mode	Mode difference set 1/set 2
0.67	2vb1	1ucs	124.8	178.4	152.9	149.0	130.7	
0.670.76	1r6j	1yk4	88.1	253.0	133.5	109.1	96.8	
0.760.87	1m40	1n55	50.6	180.7	81.7	79.8	71.7	0.01
0.871.00	2gkg	2rbk	39.0	106.1	57.0	58.4	53.1	0.06
1.001.14	2ofm	1rqw	26.2	113.1	45.2	42.8	39.3	0.16
1.141.31	2qj7	2dlb	18.9	90.7	31.9	31.3	29.1	0.00
1.311.50	1o6v	2ew0	12.8	54.3	21.8	22.9	21.5	0.00
1.501.72	2omq	2dga	8.1	56.6	15.8	16.8	15.9	0.00
1.721.97	3ins	2egx	5.6	40.5	11.8	12.3	11.8	0.02
1.972.25	1e0p	1zba	3.8	292.5	9.1	9.0	8.7	0.00
2.252.58	2ins	2izw	2.8	565.7	7.0	6.6	6.5	0.00
2.582.95	2p3c	1ng0	2.4	465.2	6.1	4.8	4.8	0.02
2.953.37	2vdt	1dwn	1.8	694.6	7.4	3.5	3.5	0.00
3.373.86	2dc3	1c8h	1.5	293.1	8.7	2.6	2.6	0.00
3.864.42	2gsz	1x35	1.1	73.0	3.9	1.9	1.9	0.01
4.425.06	1ye1	2g34	0.9	89.5	12.7	1.4	1.4	
5.065.80	3b5x	2gp1	8.1	32.2	16.2	1.0	1.1	
5.806.63	2zqp	2zqp	0.8	0.8	0.8	0.7	0.8	
6.637.59	3c4y	1yv0	0.3	0.6	0.5	0.5	0.6	
7.598.69	2dh1	2dh1	2.6	2.6	2.6	0.4	0.4	
8.699.95	1vcr	2qzv	3.2	14.1	8.6	0.3	0.3	

**Table 3:** Coefficients of the linear approximations Each function *f*(*d*) is presented as a linear function of the resolution logarithm, *f*(*d*) = *a*ln*d* + *b*. Data sets (column 2) are defined in the text. Column 3 shows the resolution interval used to calculate the linear interpolation. Columns 6 and 8 show the root-mean-square-deviation values for the interpolation and extrapolation intervals.

Function* f*(*d*)	Data set	Interpolation interval	*a*	*b*	R.m.s.d. interpolation	Extrapolation interval	R.m.s.d. extrapolation
ln(*N*_ref_/*N*_at_)	2	0.762.58	2.31	3.91	0.0413	0.764.42	0.4503
ln[(*N*_ref_/*N*_at_)]	2	0.762.58	2.23	3.85	0.0490	0.764.42	0.0884
ln[(*N*_ref_/*N*_at_)]	2	0.762.58	2.23	3.85	0.0490	0.765.06	0.1031
ln[**(*N*_ref_/*N*_at_)]	2	0.764.42	2.25	3.83	0.0701	0.765.06	0.0732
ln[**(*N*_ref_/*N*_at_)]	2	0.764.42	2.25	3.83	0.0701	0.762.58	0.0580
ln[**(*N*_ref_/*N*_at_)]	3	0.873.37	2.10	3.68	0.0910		
*R*_PDB_	4	0.873.86	0.0874	0.1386	0.0065	0.765.06	0.0125
*R*_PDB_	4	0.765.06	0.0992	0.1339	0.0102	0.6010.0	0.0249
(*R*_PDB_)	4	0.873.86	0.0912	0.1343	0.0098	0.765.06	0.0109
(*R*_PDB_)	4	0.765.06	0.0943	0.1306	0.0107		
(*R*)	1	0.873.86	0.0716	0.1560	0.0076	0.765.06	0.0088
(*R*)	1	0.765.06	0.0695	0.1599	0.0085		
(*R*)	5	0.873.86	0.0804	0.1470	0.0070		
(*R*_free_)	5	0.873.86	0.1050	0.1672	0.0069		
(*R*_free_ *R*)	5	0.872.95	0.0238	0.0201	0.0022		
(*R*_PDBmin_)	4	0.602.95	0.0163	0.0884	0.0089		
(*R*_PDBmin_)	4	2.956.63	0.2859	0.2006	0.0118		

**Table 4:** Statistical information for the *R* factors in resolution shells chosen uniformly on the logarithmic scale Columns 2, 3 and 4 give the PDB codes for the models with the minimal *R*-factor values reported in the PDB headers and recalculated by *phenix.model_vs_data* (mvd). Linear interpolations are given for the mode of corresponding values calculated for sets 4 (column 5) and set 5 (columns 68).

	PDB code			Linear interpolation			
Resolution shell ()	Min. *R* (PDB)	Min. *R* (mvd, set 4)	Min. *R* (mvd, set 5)	(*R*_PDB_)	(*R*)	(*R*_free_)	(*R*_free_ *R*)
0.67	2vb1	2vb1		0.0911	0.1090	0.1175	0.0089
0.670.76	1yk4	1r6j	2pve	0.1035	0.1199	0.1317	0.0121
0.760.87	2ol9	2h5c	2h5c	0.1158	0.1307	0.1459	0.0153
0.871.00	1ob7	1rb9	1ixb	0.1281	0.1416	0.1601	0.0185
1.001.14	1iro	1iro	1z3n	0.1405	0.1525	0.1742	0.0217
1.141.31	2v9l	1n0q	2v9l	0.1528	0.1634	0.1884	0.0250
1.311.50	1hbz	2plz	1hbz	0.1651	0.1743	0.2026	0.0282
1.501.72	2ah2	6rxn	2pfg	0.1775	0.1851	0.2168	0.0314
1.721.97	1amk	2dya	2dya	0.1898	0.1960	0.2310	0.0346
1.972.25	2oh5	2oh5	2oh5	0.2021	0.2069	0.2452	0.0378
2.252.58	2oh7	1uvw	1uvw	0.2145	0.2178	0.2594	0.0410
2.582.95	5bna	1tre	1f4h	0.2268	0.2286	0.2736	0.0443
2.953.37	1bgj	1sv2	1ydz	0.2391	0.2395	0.2878	0.0475
3.373.86	2d3b	1gn3	2q3n	0.2515	0.2504	0.3020	0.0507
3.864.42	1aos	1veq	1veq	0.2638	0.2613	0.3162	0.0539
4.425.06	2rkj	1pgf	2rkj	0.2761	0.2721	0.3304	0.0571
5.065.80	3b5w	2b66	3b5x	0.2885	0.2830	0.3445	0.0603
5.806.63	2b9n	2b9n	3e3j	0.3008	0.2939	0.3587	0.0635
6.637.59	3c4y	3c4y	1yv0	0.3131	0.3048	0.3729	0.0668
7.598.69	2dh1	2dh1	2dh1	0.3255	0.3157	0.3871	0.0700
8.699.95	1vcr	1zbb	1vcr	0.3378	0.3265	0.4013	0.0732

## References

[bb1] Adams, P. D., Grosse-Kunstleve, R. W., Hung, L.-W., Ioerger, T. R., McCoy, A. J., Moriarty, N. W., Read, R. J., Sacchettini, J. C., Sauter, N. K. & Terwilliger, T. C. (2002). *Acta Cryst.* D**58**, 1948–1954. 10.1107/s0907444902016657 12393927

[bb3] Afonine, P. V., Grosse-Kunstleve, R. W. & Adams, P. D. (2005). *Acta Cryst.* D**61**, 850–855. 10.1107/S0907444905007894 PMC2808320 15983406

[bb4] Afonine, P. V., Grosse-Kunstleve, R. W., Adams, P. D., Lunin, V. Y. & Urzhumtsev, A. (2007). *Acta Cryst.* D**63**, 1194–1197. 10.1107/S0907444907046148 PMC2808317 18007035

[bb2] Afonine, P. V., Lunin, V. Y., Muzet, N. & Urzhumtsev, A. (2004). *Acta Cryst.* D**60**, 260–274. 10.1107/S0907444903026209 14747702

[bb5] Arai, S., Chatake, T., Suzuki, N., Mizuno, H. & Niimura, N. (2004). *Acta Cryst.* D**60**, 1032–1039. 10.1107/S0907444904006559 15159562

[bb6] Bernstein, F. C., Koetzle, T. F., Williams, G. J., Meyer, E. F. Jr, Brice, M. D., Rodgers, J. R., Kennard, O., Shimanouchi, T. & Tasumi, M. (1977). *J. Mol. Biol.* **112**, 535–542. 10.1016/s0022-2836(77)80200-3 875032

[bb7] Berman, H. M., Westbrook, J., Feng, Z., Gilliland, G., Bhat, T. N., Weissig, H., Shindyalov, I. N. & Bourne, P. E. (2000). *Nucleic Acids Res.* **28**, 235–242. 10.1093/nar/28.1.235 PMC102472 10592235

[bb8] Brändén, C.-I. & Jones, T. A. (1990). *Nature (London)*, **343**, 687–689.

[bb9] Brünger, A. T. (1992). *Nature (London)*, **355**, 472–475. 10.1038/355472a0 18481394

[bb10] Brünger, A. T. (1997). *Methods Enzymol.* **276**, 366–396. 10.1016/s0076-6879(97)77021-6 18488318

[bb11] Cruickshank, D. W. J. (1996). *Proceedings of the CCP4 Study Weekend. Refinement of Macromolecular Structures*, edited by E. Dodson, M. Moore, A. Ralph & S. Bailey, pp 11–22. Warrington: Darebury Laboratory.

[bb12] Fabiola, F., Korostelev, A. & Chapman, M. S. (2006). *Acta Cryst.* D**62**, 227–238. 10.1107/S0907444905040680 16510969

[bb13] Howard, E. I., Sanishvili, R., Cachau, R., Mitschler, A., Chevrier, B., Barth, P., Lamour, V., Van Zandt, M., Sibley, E., Bon, C., Moras, D., Schneider, T. R., Joachimiak, A. & Podjarny, A. (2004). *Proteins*, **55**, 792–804. 10.1002/prot.20015 15146478

[bb14] Jiang, J.-S. & Brünger, A. T. (1994). *J. Mol. Biol.* **243**, 100–115. 10.1006/jmbi.1994.1633 7932732

[bb15] Joosten, R. P. *et al.* (2009). *J. Appl. Cryst.* **42**, 376–384. 10.1107/S0021889809008784 PMC3246819 22477769

[bb16] Kantardjieff, K. A. & Rupp, B. (2003). *Protein Sci.* **12**, 1865–1871. 10.1110/ps.0350503 PMC2323984 12930986

[bb17] Lamour, V., Barth, P., Rogniaux, H., Poterszman, A., Howard, E., Mitschler, A., Van Dorsselaer, A., Podjarny, A. & Moras, D. (1999). *Acta Cryst.* D**55**, 721–723. 10.1107/s0907444998013365 10089480

[bb18] Luzzati, V. (1952). *Acta Cryst.* **5**, 802–810.

[bb19] Matthews, B. W. (1968). *J. Mol. Biol.* **33**, 491–497. 10.1016/0022-2836(68)90205-2 5700707

[bb20] Morris, R. J., Blanc, E. & Bricogne, G. (2004). *Acta Cryst.* D**60**, 227–240. 10.1107/S0907444903025538 14747698

[bb21] Morris, R. J. & Bricogne, G. (2003). *Acta Cryst.* D**59**, 615–617. 10.1107/s090744490300163x 12595742

[bb22] Pichon-Pesme, V., Lachekar, H., Souhassou, M. & Lecomte, C. (2000). *Acta Cryst.* B**56**, 728–737. 10.1107/s0108768100004390 10944266

[bb23] Read, R. J. & Kleywegt, G. J. (2009). *Acta Cryst.* D**65**, 140–147. 10.1107/S0907444908041085 PMC2631636 19171969

[bb24] Schäfer, M., Sheldrick, G. M., Bahner, I. & Lackner, H. (1998). *Angew. Chem.* **37**, 2381–2384. 10.1002/(SICI)1521-3773(19980918)37:17<2381::AID-ANIE2381>3.0.CO;2-L 29710967

[bb25] Tickle, I. J., Laskowski, R. A. & Moss, D. S. (1998). *Acta Cryst.* D**54**, 547–557. 10.1107/s0907444997013875 9761849

[bb26] Tickle, I. J., Laskowski, R. A. & Moss, D. S. (2000). *Acta Cryst.* D**56**, 442–450. 10.1107/s0907444999016868 10739917

[bb27] Urzhumtseva, L., Afonine, P. V., Adams, P. D. & Urzhumtsev, A. (2009). *Acta Cryst.* D**65**, 297–300. 10.1107/S0907444908044296 PMC2651759 19237753

[bb28] Wilson, A. J. C. (1949). *Acta Cryst.* **2**, 318–321.

[bb29] Zwart, P. H., Grosse-Kunstleve, R. W. & Adams, P. D. (2005). *CCP4 Newsl.* **43**, contribution 7.

